# Impact of COVID-19 on the Self-Report Assessment of Obsessive-Compulsive Disorder

**DOI:** 10.7759/cureus.58457

**Published:** 2024-04-17

**Authors:** Elizabeth Meza, Gabriel Hapenciuc, Michelle A Philip, Janet T Le, Ryan J Marek

**Affiliations:** 1 Primary Care and Clinical Medicine, Sam Houston State University College of Osteopathic Medicine, Conroe, USA; 2 Psychology and Philosophy, Sam Houston State University, Huntsville, USA

**Keywords:** compulsion, ocd and related disorder, mmpi-3, obsessive compulsive disorders, covid-19

## Abstract

Background

The COVID-19 pandemic and subsequent guidelines have had a substantial effect on social norms. This likely affected self-report assessment of psychopathology, namely those that assess obsessive-compulsive tendencies routinely used to screen for obsessive-compulsive disorder (OCD). It was hypothesized that self-report assessment of OCD likely produces inflated, non-discriminating scale scores.

Methods

Data collection occurred prior to the COVID-19 pandemic with the aim of validating a new psychological test; however, data collection was abruptly halted in March 2020. Data collection was allowed to resume in the latter half of the year. Both groups were racio-ethnically and gender diverse.

Results

Self-report measures of OCD yielded inflated scores. For instance, the total obsessive-compulsive inventory-revised (OCI-R) average score of all participants went from normative levels prior to COVID-19 (M = 13.69, SD = 10.32) to an average score that was above the clinical cut-off on the OCI-R (M = 32.89; SD = 12.95) during the pandemic (t(135) = 9.66, p < 0.001, Cohen’s d = 1.66).

Conclusions

OCD-related scale scores likely produced false positives in research and practice due to COVID-19 health guidelines put in place to protect against infection that may otherwise be considered contamination fears on OCD measures.

## Introduction

SARS-CoV-2, more commonly known as COVID-19, has greatly changed the scope of human behavior since its onset. With the introduction of behaviors such as social distancing, mask mandates, and proper handwashing protocols, many people have found themselves adopting a new set of daily routines that they likely never expected to [1,2]. Because of this change in behavior, diverse research is needed to identify and quantify the impact that COVID-19 has had on the world population. Additionally, more research can be directed toward how these life-altering effects impacted those individuals who already had preexisting conditions such as anxiety, depression, and obsessive-compulsive disorder (OCD).

Once the COVID-19 pandemic began its course, researchers began investigating the individual factors that protect against infection and particular patient populations at risk of negative effects. A study by Gröndal et al. [3] found that individuals who were predisposed to irritability, anger, and impulsivity may have a slightly higher risk of experiencing the negative effects of the pandemic. They also found that emotional regulation via cognitive reappraisal potentially could guard against experiencing these negative emotions during the pandemic. Other investigations into the effects of the COVID-19 pandemic found physical and mental well-being, functionality, and behavior were negatively impacted. A study by Borbás et al. looked at the mental health of both adult and pediatric patients during the early months of the pandemic [4]. In this study, emphasis was given to both patient behavior and neuroimaging (particularly through the use of functional magnetic resonance imaging, or fMRI). Analysis of the neuroimaging results found that activation of the right prefrontal cortex prior to the onset of the COVID-19 pandemic was correlated with the development of a fear of becoming ill. Contrarily, activation of the right temporoparietal junction prior to the onset of the pandemic did not correlate to the development of a fear of becoming ill. With regards to psychopathology, 32.56% of adults presented with elevated scores for depression, and 34.88% with clinically elevated scores for anxiety. Children showed no significant change in behavior, but their self-reported mood was correlated with the depression scores of their mothers. Additionally, the children whose mothers self-reported feeling higher levels of burden of caregiving were found to have more emotional and behavioral problems [4].

The levels of anxiety and depression because of the pandemic have varied based on the individual's method of coping. For instance, Türk et al. focused on analyzing the impact of COVID-19 on levels of depression and anxiety [5]. The study utilized various Likert scales to screen for six different psychopathologies: separation anxiety disorder, generalized anxiety disorder, panic disorder, social phobia, OCD, and major depressive disorder. More than half of adolescents in the sample had high levels of depression and anxiety relative to mean scores across these domains within one standard deviation. It was also found that these levels were higher in high school-aged adolescents and women. Many of these adolescents had an increase in the severity of symptoms when consuming social media (particularly social media related to the pandemic). Additionally, different coping strategies were found to have differing effects on levels of depression and anxiety. Participants with active coping strategies (those that focus on solving problems and functionality) tended to score lower on depression and anxiety when compared to participants with avoidant (a non-functional style that avoids solving the problem at hand) and negative (exaggerating the scale of the problem and placing blame on themselves and others, as well as performing risky behaviors) coping styles.

In Koch’s 2021 review [6] of the impact of COVID-19 on anxiety disorders, an increased prevalence of anxiety-related symptoms was found in the general population during the COVID-19 pandemic. In addition, patients with a diagnosis of OCD had obsessions and compulsions that were related to the COVID-19 pandemic. It was also found that the pandemic did not have a significant impact on the symptoms of patients diagnosed with social phobia [6].

Van Ameringen et al. highlight the profound impact the COVID-19 pandemic had on individuals with OCD. Individuals with OCD were especially susceptible to symptom exacerbation and relapse because of messaging and restrictions related to the pandemic [7]. The International College of Obsessive-Compulsive Spectrum Disorders (ICOCS) released guidelines for OCD during COVID-19 that focus on helping clinicians practice a more nuanced treatment approach [7]. Similarly, Jelinek et al. observed an increase in the severity of reported OCD symptoms, total OCI-R score positively correlated with the increased severity, and dysfunctional ideas about hygiene that were associated with an increase in OCD symptoms. These were especially specific to patients with compulsions related to handwashing. Patient Health Questionnaire-9 (PHQ-9) self-report data was also utilized, and it was found that patients also had moderate levels of self-reported depression scores on the PHQ-9 [8].

According to Hansen et al., asking individuals to self-report the frequency of routine behaviors during the time of the COVID-19 pandemic introduced significant measurement errors and systematic biases [9]. Although self-reported behavior is convenient to obtain, research has suggested that people cannot accurately report routine behaviors. Therefore, the self-reports of COVID-19 hygiene-relevant routine behaviors are not necessarily valid measures of actual behavioral frequencies. Hansen et al. emphasized that when feasible, using human observers to collect actual behavioral data is preferable to self-reported behavior [9].

Because the COVID-19 pandemic had a remarkable effect on the measurement and self-reporting of psychopathologies in different countries, it is likely that the scientific community must now address both cutoff scores for measuring psychopathologies and for assessing whether current measures (such as the MMPI-3) have been statistically impacted by COVID-19. The scope of this paper is to address these concerns for the diagnosis of OCD and help ensure that we are minimizing both false positives and false negatives to ensure that patients are getting clinically accurate diagnoses when utilizing the MMPI-3 for screening patients.

It was hypothesized that self-report assessment of OCD would subsequently produce inflated, non-discriminating scale scores in the sample collected during the pandemic.

## Materials and methods

Participants

These data were collected for purposes of examining the MMPI-3 Compulsivity (CMP) scale with external criteria. Data collection started prior to COVID-19 (November 2019-March 2020) and was immediately suspended due to the 2020 COVID-19 pandemic. Data collection was allowed to resume from September 2020 to December 2020, creating an unintentional opportunity to evaluate the impact of the pandemic on the self-report of OCD symptoms. The use of these archival data was approved by the Sam Houston State University Institutional Review Board (approval no: IRB-2022-88) and data are available for replication purposes per request. This study was not preregistered.

Pre-COVID sample

The pre-COVID sample was composed of 86 participants who were recruited at University of Houston-Clear Lake in Houston, United States. A total of 11 participants were excluded because their MMPI-3 protocols were invalid based on criteria outlined in the MMPI-3 Technical Manual [10]: cannot say (CNS) ≥ 15, combined response inconsistency (CRIN) ≥ 80T, variable response inconsistency (VRIN) ≥ 80T, true response inconsistency (TRIN) ≥ 80T, infrequent responses (F) = 100T, and infrequent psychopathology responses (Fp) ≥ 100T. A total of 75 participants were retained for further analyses. Of the remaining sample, 73.2% (55) were women and 26.8% (20) were men. The mean age of participants was 26.8 years old (SD = 9.8; range 18-63). The overall sample was racio-ethnically diverse: 42.7% Caucasian, 25.3% Latinx, 14.7% Multiracial, 6.7% African-American, and 10.6% composed of other ethnicities.

Pandemic sample

An independent pandemic sample was composed of 70 participants who were recruited at the same southern university in the southern United States. A total of six participants were excluded because their MMPI-3 protocols were invalid based on criteria outlined in the MMPI-3 Technical Manual [10]. A total of 64 participants were retained for further analysis. Of the remaining sample, 20.0% (14) were men, 77.1% (54) were women, and 2.9% (2) reported another gender identity. The mean age of participants was 23.86 years old (SD = 6.72; range 18-55). The overall sample was racio-ethnically diverse with 21.9% Caucasian, 25.0% Latinx, 23.4% Multiracial, 17.2% African-American, and 12.5% composed of other ethnicities.

Measures

Minnesota Multiphasic Personality Inventory-3

The Minnesota Multiphasic Personality Inventory-3 (MMPI-3) [11,12] was scored from a version of the MMPI-2-Restructured Form using an expanded item pool (MMPI-2-RF-EX). The MMPI-3 contains (335) true/false items and is scored across 10 validity scales and 42 substantive scales that assess a wide range of psychopathology and personality in accordance with contemporary models of psychopathology [13]. The test was standardized using a sample that represents projected 2020 census norms. The MMPI-3 Technical Manual lists reliability and validity coefficients for all scale scores across numerous samples (normative sample, undergraduate sample, various mental health samples, medical samples, and forensic samples). Only the CMP scale was used in this study. The internal consistency of the CMP scale in the pre-pandemic and pandemic samples were 0.67 and 0.67, respectively.

Obsessive-Compulsive Inventory-Revised

The obsessive-compulsive inventory-revised (OCI-R) [14] is an 18-item, self-report measure that assesses the symptomology of OCD. Item content related to OCD symptoms includes washing, checking, ordering, hoarding, neutralizing, and obsessing. The items are summed, resulting in a range of scores between 0 and 72 with the measure yielding good psychometric properties in other samples [13]. Internal consistency of the OCI-R scale in the pre-pandemic and pandemic samples were 0.87 and 0.93, respectively.

Florida Obsessive-Compulsive Inventory

The Florida Obsessive-Compulsive Inventory (FOCI) [15] is a self-report measure that examines symptoms and severity of OCD. The measure yields good psychometric properties, including convergent validity [14]. The first 24 items are screening items. If any of the first 24 items are endorsed, part B (five items) is then administered. For this study, all items from part B were administered to participants because they reflect a dimensional OCD severity scale. Internal consistency of the FOCI-B scale in the pre-pandemic and pandemic samples were 0.90 and 0.92, respectively.

Dimensional Obsessive-Compulsive Scale

The Dimensional Obsessive-Compulsive Scale (DOCS) [16] is a 20-item, self-report measure that assesses four obsessive-compulsive symptoms dimensions: contaminating/washing, harm obsessions/checking compulsions, symmetry/ordering, and unacceptable thoughts. The measure yields good psychometric properties and is dimensional in nature to assess severity. Internal consistency of the DOCS scale in the pre-pandemic and pandemic samples were 0.91 and 0.93, respectively.

Inventory of Depression and Anxiety Symptoms-II

The Inventory of Depression and Anxiety Symptoms-II (IDAS-II) [17] is a 99-item, self-report broadband measure that assesses mood and anxiety-related symptoms. The items of the IDAS-II can be scored across various scales pertaining to depression (general depression, dysphoria, suicidality, lassitude, insomnia, appetite loss, appetite gain), anxiety (panic, social anxiety, traumatic intrusions, claustrophobia, traumatic avoidance, checking, ordering, cleaning) and bipolar (mania, Euphoria). The scales of the IDAS-II yield good psychometric properties, notably reliability, and convergent validity coefficients [17,18]. The checking, ordering, and cleaning scales were used in the current investigation. Internal consistency of the IDAS scales for the checking, ordering, and cleaning scales in the pre-pandemic and pandemic samples was 0.81, 0.64, 0.91 and 0.86, 0.89, and 0.88, respectively.

Procedure

Participants completed the study in small groups in exchange for course credit. Once informed consent was obtained, participants were administered the MMPI-2-RF-EX followed by collateral questionnaires. The pre-COVID sample was collected in person with a proctor present. The pandemic sample was administered the measures online via a face-to-face platform with a proctor in accordance with guidelines for telehealth assessment [10].

Statistical analysis

Means and standard deviations for all scale scores used in this study were calculated and reported in Table 1 between samples and by gender. An overall multiple analysis of variance (MANOVA) was calculated using the eight dependent variables. Two independent variables were added to the model: method (pre-pandemic and pandemic) and gender (male and female). A MANOVA was calculated to protect against type 1 errors. Following the MANOVA, factorial analysis of variances (ANOVAS) was calculated to observe whether or not self-reported OCD-related measures were statistically higher during the pandemic compared to the pre-pandemic scores, whether any statistically significant gender differences emerged, and whether there were interaction effects between gender and the samples on OCD-related criteria.

**Table 1:** Means and standard deviations for OCD data OCD: Obsessive-compulsive disorder; MMPI-3 CMP: Minnesota Multiphasic Personality Inventory-3 compulsivity scale; OCI-R: Obsessive-Compulsive Inventory-Revised; FOCI: Florida Obsessive-Compulsive Scale; DOCS: Dimensional Obsessive-Compulsive Scale; IDAS: Inventory for depression and anxiety symptoms; SD: Standard deviation

	Non-COVID				COVID			
	Men (n = 20)		Women (n = 55)		Men (n = 14)		Women (n = 54)	
	Mean	SD	Mean	SD	Mean	SD	Mean	SD
MMPI-3 CMP	55T	9	54T	10	61T	9	59T	10
OCI-R	15.84	11.62	15.79	14.23	34.57	13.32	35.42	15.29
FOCI part A	3.89	4.61	4.00	4.49	35.86	3.23	34.32	4.71
FOCI part B	3.06	2.84	3.12	3.77	9.73	5.44	11.75	4.90
DOCS	8.16	9.26	8.31	9.15	40.79	13.02	43.43	15.74
IDAS ordering	1.56	0.48	1.48	0.59	2.14	0.60	1.97	1.01
IDAS cleaning	1.52	0.74	1.40	0.69	1.95	0.60	2.07	1.00
IDAS checking	1.80	1.24	1.63	0.77	2.40	1.04	2.32	1.07

## Results

Descriptive statistics consisting of mean scale scores and standard deviations were calculated for the MMPI-3 CMP, OCIR total, FOCIA, FOCIB, DOCS total, IDAS ordering, IDAS cleaning, and IDAS checking scale scores broken down by method (pre-pandemic and pandemic) and gender (male vs. female). These descriptive statistics are reported in Table 1 whereas the inferential statistics for these comparisons are listed in Table 2. By and large, mean scale scores were higher in the pandemic sample than the pre-pandemic sample; however, scale scores appeared to be similar for both men and women in both samples.

**Table 2:** Inferential statistics for OCD data OCD: Obsessive-compulsive disorder; MMPI-3 CMP: Minnesota Multiphasic Personality Inventory-3 compulsivity scale; OCI-R: Obsessive-Compulsive Inventory-Revised; FOCI: Florida Obsessive-Compulsive Scale; DOCS: Dimensional Obsessive-Compulsive Scale; IDAS: Inventory for depression and anxiety symptoms

		F (df)	p-value	Partial ╖^2^
	Method	4.55 (1, 129)	0.035*	0.034
MMPI-3 CMP	Gender	0.43 (1, 129)	0.515	0.003
	Interaction	0.93(1, 129)	0.930	<0.001
	Method	56.37 (1, 127)	<0.001	0.307
OCI-R	Gender	0.132 (1, 127)	0.717	0.001
	Interaction	0.713 (1, 127)	0.400	0.006
	Method	1386.56 (1, 126)	<0.001	0.917
FOCI part A	Gender	1.33 (1, 126)	0.252	0.010
	Interaction	0.151 (1, 126)	0.698	0.001
	Method	57.14 (1, 104)	<0.001	0.355
FOCI part B	Gender	2.46 (1, 104)	0.120	0.023
	Interaction	3.28 (1, 104)	0.073	0.031
	Method	187.96 (1, 128)	<0.001	0.595
DOCS	Gender	0.163 (1, 128)	0.687	0.001
	Interaction	0.918 (1, 128)	0.340	0.007
	Method	11.91 (1, 126)	<0.001	0.086
IDAS ordering	Gender	1.20 (1, 126)	0.276	0.009
	Interaction	0.002 (1, 126)	0.961	<0.001
	Method	12.22 (1, 126)	<0.001	0.088
IDAS cleaning	Gender	0.162 (1, 126)	0.688	0.001
	Interaction	0.943 (1, 126)	0.333	0.007
	Method	11.10 (1, 126)	0.001*	0.081
IDAS checking	Gender	0.513 (1, 126)	0.475	0.004
	Interaction	0.575 (1, 126)	0.450	0.005

Results of the MANOVA suggested that the higher scores on the OCD measures observed in the pandemic sample were statistically and significantly higher than the pre-pandemic sample overall (Pillai’s Trace F (8, 95) = 161.38, p < 0.001, partial η2 = 0.93). Overall, MANOVA tests suggested there were no overall gender effects (Pillai’s Trace F (8, 95) = 1.253, p = 0.277, partial η2 = 0.10) or interaction effects (Pillai’s Trace F (8, 95) = 1.076, p = 0.387, partial η2 = 0.08). Two by two factorial ANOVAs for each of the dependent variables are reported in Table 2. Across all dependent variables, OCD scale scores were statistically higher in the pandemic sample compared to the pre-pandemic sample, yielding modest to large effect sizes.

## Discussion

While collecting data to evaluate the effectiveness of a new Specific Problems Scale of the MMPI-3, the effects of the COVID-19 pandemic halted data collection. When data collection resumed in the latter half of 2020 during the pandemic, it was hypothesized that self-report assessment of OCD would subsequently produce inflated, non-discriminating scale scores in the sample collected during the pandemic. Statistical analysis of the self-assessment OCD data collected before and during the pandemic supports the hypothesis of inflated scores due to statistically significant higher scores across OCD-related scale scores in the pandemic sample as compared to the pre-COVID sample. The evaluation also controlled for stress, and the results continued to demonstrate a statistically significant increase in average scale scores. These scale scores were shown to be non-discriminating as inferential statistics demonstrated non-significant differences between genders.

The impact that the COVID-19 pandemic had on self-reported measures of psychopathology needs to be better understood. As outlined earlier, many guidelines suggested and promoted behaviors that would normally be considered for a contamination-specific type of OCD. As suspected, statistically significant higher scores across OCD-related scale scores in the pandemic sample as compared to the pre-COVID sample. In fact, the means and standard deviations for some scale scores, such as the OCI-R, were well above the recommended cut-scores for screening positive for possible OCD in the pandemic sample. This indeed likely affected clinical practice during the pandemic where these symptom/screener-based measures were used. They likely produced unusually high false positive rates.

Another confounding variable could be what is termed "cyberchondria" [19,20]. Cyberchondria is the persistent and compulsive act of searching for health information online, which can lead to heightened health anxiety and distress. This transdiagnostic compulsive behavioral syndrome is driven by both anxiety and compulsive tendencies. Correlates of persons who are higher in cyberchondria include greater health anxiety, excessive internet use, and more symptoms of OCD. To some extent, cyberchondria could be driving the greater variability in scores during the pandemic - notably when there were no treatment options for COVID-19.

One of the limitations of the study was the lack of structured interviews to accompany the self-report assessment scale scores. The implementation of a structured interview to diagnose OCD in both the pre-COVID sample and the pandemic sample, in conjunction with the self-report assessment results would allow us to conclusively determine whether the scores were inflated or if the incidence of OCD increased during the pandemic. Although it is unlikely that the incidence of OCD in the intra-COVID sample would rapidly cross the OCR-I threshold as a byproduct of substantial OCD changes, this could be a future research direction. Another limitation of this study is that there is no continuity between the pre-COVID and current population samples because the randomized participants were not screened a second time to compare the effects of the pandemic on OCD. Therefore, further studies should elucidate the long-term effects of COVID-19 or other stressors on the accuracy of OCD screening tools. This study was also limited in the available research on OCD, psychopathology, and mental health within the United States during the pandemic. The available studies conducted in European countries investigated the negative effects of COVID-19, COVID-era social media, and the emergence of a new phobia related specifically to the COVID-19 pandemic, yet there is little to no research on OCD during the pandemic specifically. Additionally, many of these studies utilized self-report online surveys as opposed to structured interviews or behavior observation by researchers to discern changes in mental health status and psychopathology as a result of the pandemic. Further studies that focused on the impact of the pandemic on individuals with OCD that also utilized structured interviews or behavior observation by researchers would provide a more accurate measure of whether there was an increase in the incidence of OCD as a result of the pandemic or if the scale scores were artificially inflated, resulting in false positive results.

## Conclusions

The pandemic has affected the world in many ways, one of the biggest being mental health. With all of the changes, it is important to evaluate whether the current screening tools for psychopathology remain accurate. This study specifically provides new insight into the screening tools available for OCD and how it was affected by the pandemic. A future direction of this project could be to observe regional differences in self-report assessment scores and whether the same inflation is seen among populations that hold varying attitudes toward COVID-19. Further research may be necessary to evaluate whether adjusting the cutoff score for OCD screening will more accurately assess OCD behavior post-pandemic. Additionally, given the inflated scores pertaining to OCD, it would be beneficial to see how other psychopathologies have been affected by the pandemic (i.e., was there an increase in psychopathology during this time or were high scores on these measures impacted by health guidelines, and COVID-19 (e.g., low energy, sleeping more/less)).
